# Hybrid gait training with an overground robot for people with incomplete spinal cord injury: a pilot study

**DOI:** 10.3389/fnhum.2014.00298

**Published:** 2014-05-13

**Authors:** Antonio J. del-Ama, Ángel Gil-Agudo, José L. Pons, Juan C. Moreno

**Affiliations:** ^1^Biomechanics and Technical Aids Unit, National Hospital for Spinal Cord InjuryToledo, Spain; ^2^Bioengineering Group, Spanish National Research CouncilMadrid, Spain

**Keywords:** lower extremity, gait, spinal cord injury, hybrid exoskeleton, muscular electrical stimulation, motor recovery

## Abstract

Locomotor training has proved to provide beneficial effect in terms of mobility in incomplete paraplegic patients. Neuroprosthetic technology can contribute to increase the efficacy of a training paradigm in the promotion of a locomotor pattern. Robotic exoskeletons can be used to manage the unavoidable loss of performance of artificially driven muscles. Hybrid exoskeletons blend complementary robotic and neuro-prosthetic technologies. The aim of this pilot study was to determine the effects of hybrid gait training in three case studies with persons with incomplete spinal cord injury (iSCI) in terms of locomotion performance during assisted gait, patient-robot adaptations, impact on ambulation and assessment of lower limb muscle strength and spasticity. Participants with iSCI received interventions with a hybrid bilateral exoskeleton for 4 days. Assessment of gait function revealed that patients improved the 6 min and 10 m walking tests after the intervention, and further improvements were observed 1 week after the intervention. Muscle examination revealed improvements in knee and hip sagittal muscle balance scores and decreased score in ankle extensor balance. It is concluded that improvements in biomechanical function of the knee joint after the tested overground hybrid gait trainer are coherent with improvements in gait performance.

## INTRODUCTION

Locomotor training has proved to provide beneficial effect in terms of mobility in incomplete paraplegic patients. Improvement of locomotor activity occurs independently of the spontaneous recovery of the spinal cord function. With training functional movements under close physiological conditions, sensory inputs and central neuronal circuits become activated, with important spinal cord regeneration effects. Such training paradigm of stepping that relies on the entrainment of control of movement by driving the limbs through trajectories can be implemented with wearable actuated exoskeletons.

A further training approach is the use of EMS to activate paralyzed muscles for inducing gait. The importance of the EMS-induced gait training approach emerges from the demonstrated benefits it provides to the patient, mainly related to muscle strength and cardiorespiratory fitness (Creasey et al., 2004; Nightingale et al., 2007; Graupe et al., 2008; Lam et al., 2010). Besides, EMS can contribute to the stimulation of sensory input from the muscle that may be beneficial in case of damage. Nevertheless, it is not so effective yet in gait recovery (Thrasher et al., 2006), due to muscle fatigue, rapidly induced by EMS, leading to interruptions in training. The combination of a robotic exoskeleton with an EMS system results in a Hybrid Exoskeleton (del-Ama et al., 2012c). Hybrid exoskeletons posses several advantages for implementing novel alternatives of gait training. Firstly, hybrid exoskeletons takes advantage of the fine control of joint trajectories and the ability of delivering power that can compensate the poor quality of EMS-induced joint movement (Goldfarb and Durfee, 1996). On the other hand, muscle power generated by EMS can reduce the energy demand of the exoskeleton, thereby requiring less powerful joint actuators, which would result in a lighter system. Secondly, hybrid exoskeletons may promote more effective neural plasticity than other standard practices like treadmill training, because of the intensive, community-based gait practice involved. This gait practice occurs during daily training, and thus, increased user participation is promoted during walking training.

Designing a control strategy that adequately manages muscle fatigue is crucial to the development of a successful hybrid exoskeleton that can provide longer periods of training. Muscle fatigue caused by EMS is critical in cases of muscle atrophy, which are typically found in the SCI population. Effective closed-loop control of EMS has been proposed to manage muscle performance based on the kinetics of human–robot interaction (Stauffer et al., 2009; del-Ama et al., 2012d). Besides, closed-loop control of joint movement would be required to counteract the effect of muscle fatigue (Goldfarb et al., 2003). An extensive review of control approaches and management of muscle fatigue of hybrid exoskeletons is reported in (del-Ama et al., 2012b).

While various wearable exoskeletons were successful in achieving gait in subjects with spinal cord injuries (Dollar and Herr, 2007, 2008; Hesse et al., 2010), this has generally been proposed as a functional substitution. An automatic treatment combining the AAN and cooperative-control principles, using state-of-the art hybrid technologies, could produce feasible systems in which the robot handles efficient delivery of EMS-induced torque. Besides, AAN control strategies of hybrid NP and robotic systems must work in parallel with the human system. These systems are likely to achieve sustained training sessions with EMS if incorporating techniques to control the appearance of muscle fatigue (del-Ama et al., 2012a).

Kinesis is a hybrid robotic device that has been developed for overground gait training in incomplete spinal cord injuries. The objective of this study is to evaluate the performance of Kinesis and the hybrid-cooperative walking therapy within the target population. In a previous study we conducted the evaluation of the effect of hybrid control of walking in a group of healthy subjects, demonstrating the feasibility of the ambulatory hybrid exoskeleton to effectively balance robotic and EMS during walking in intact humans (del-Ama et al., 2013). Based on that, we propose the current pilot study for validation of the hybrid-cooperative control approach. A comprehensive protocol was for evaluation of the impact on the walking function of the patient.

The design of this pilot clinical evaluation comprises pre–post assessment of the intervention effect in walking function of the patients. In particular, the design follows two objectives: (1) to assess the direct effects on walking of gait with Kinesis, focusing on control aspects and Kinesis-patient mutual adaptations; (2) to assess the impact of training with Kinesis on the ambulation of a sample of incomplete spinal cord injured subjects. We report the effects of hybrid gait training in three cases of persons with iSCI in terms of locomotion performance during assisted gait with and without EMS, patient-robot adaptations, impact on ambulation and assessment of lower limb muscle strength and spasticity.

## MATERIALS AND METHODS

### PARTICIPANTS

Participants with incomplete SCI (*n* = 3) were enrolled in the study to test the hybrid bilateral exoskeleton. The inclusion criteria considered patients whose lesion was categorized as conus medullaris, (injuries that affect the spinal levels among L1 and L2). The prognosis of functional recovery of walking is that these patients can walk short distances but depending on the wheelchair for community ambulation. Therefore, a successful overground hybrid walking therapy may provide benefits to this population. The functional characteristics of this lesion in relation to the walking function are: (a) preserved hip flexion ability, (b) partial ability to generate voluntary knee extension, (c) paralysis of ankle joint, and (d) presence of mild to severe spasticity.

Subject 1 is a male of 35 years old, 65 kg and 1.8 m height. He had a SCI L5 Asia impairment scale (AIS) D resulting in mild walking impairment. A secondary consequence of the lesion was impaired balance during left leg stance. Subject 2 is a male of 43 years old, 75 kg and 1.77 m height. He had a traumatic lesion, resulting in a SCI L4 AIS grade D. The patient had a passive limitation on the articular range of both knees, which caused to adapt the kinematic pattern of the left leg to meet these limits. The patient walked with fixed left knee joint during stance (at maximum extension) and a compensatory kinematic pattern for the right knee during the swing phase. Subject 3 is a male of 40 years old, 70 kg and 1.8 m height. He suffered an accident resulting in a SCI at L1 level, AIS A, with partial motor preservation at L3 level, and partial sensitive preservation at L4 level. This lesion represents the most impaired functional condition that met the inclusion criteria. The use of parallel bars was selected for the walking experimental conditions involving patient 3, given his functional status.

### HYBRID GAIT TRAINER

The Kinesis system is a bilateral wearable knee-ankle-foot orthosis, equipped with active actuators at the knee hinges and a passive elastic actuator at the ankle. FSRs are employed for monitoring floor contact and custom force sensors are available to measure human–robot interaction torques. Kinesis has a PC-controlled stimulator that delivers biphasic current-controlled rectangular pulses to knee extensor (rectus femoris and vastus lateralis) and flexor (semitendinosus and biceps femoris) muscles through surface electrodes. The controller of Kinesis is comprised by the EMS controller and the robotic controller, both managed within a high-level controller is in charge of a cooperative behavior controlling exoskeleton actuators, EMS of knee joint muscles, and management of muscle fatigue. The EMS controller is comprised by a iterative learning control algorithm for knee flexor muscles, which is only active during swing phases of gait, and a PID controller for knee extensor muscles, which is active during all the gait phases. The control task of the dual EMS controller is to minimize the interaction leg-exoskeleton forces. The robotic joints features an admittance controller, which allows to regultate the assistance of the robotic joints during walking through modulation of the stiffness of a force field imposed aroung the knee joint trajectory. Such trajectory was extracted from a normative database available in our laboratory.

The goal of the AAN controller is to reduce robotic assistance during overground ambulatory walking. To this extent, the controller is comprised by a two-state finite machine. The first state (learning state) allows the ILC to “learn” the stimulation patterns for the flexor muscles while the leg are driven by the exoskeleton. Once the algorithm has converged, the AAN controller steps into the monitoring state, where the stimulation patterns obtained in the learning state are held constant, and the assistance of the exoskeleton is modulated (increased or decrased) regarding the muscle power obtained from the EMS. This is estimated through observing the maximum angle achieved during the swing phase. If the angle exceeds 60°, the robotic assistance can be reduced, and *vice versa*.

Further details on the implementation of Kinesis EMS-robot cooperative control can be found in (del-Ama et al., 2013).

### SAFETY MEASURES

Safety measures implemented in the hybrid gait trainer included mechanical stops in the physiological limits of motion placed in ankle and knee robotic joints. In addition to this, the admittance controller of the knee joint was programmed with a software limit at maximum and minimum positions. In case of exceeding these limits, the state machine locked the motor shaft, and moved it to a default safe position. A third software safety measure consisted on the limitation of the maximum output torque required to the motor. An equivalent safe strategy was implemented in the EMS controller to set output limits for pulse width and amplitude modulation. Finally, a mechanical safety button was enabled to physically disconnect the energy supply of the entire hardware system. A risk analysis was conducted to verify the adequate actuation and response of the safety measures before actually moving to the experimental evaluation.

### PROCEDURES

**FIGURE 1:**
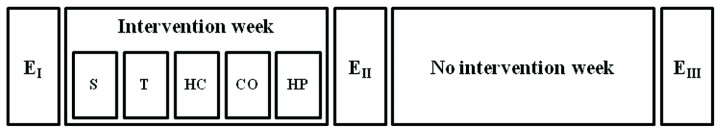
**Protocol for evaluation of hybrid gait training with Kinesis overground robot in iSCI.** Examination sessions and walking conditions are identified as follows. Walking function evaluation session: E; examination of response to EMS: S; training session: T; hybrid-cooperative condition: HC; cooperative-only condition: CO; hybrid-stiff condition: HP.

The established protocol was comprised by 2 weeks (**Figure 1**). During the first week (intervention week) several experiments related with hybrid walking training were conducted.

**FIGURE 2:**
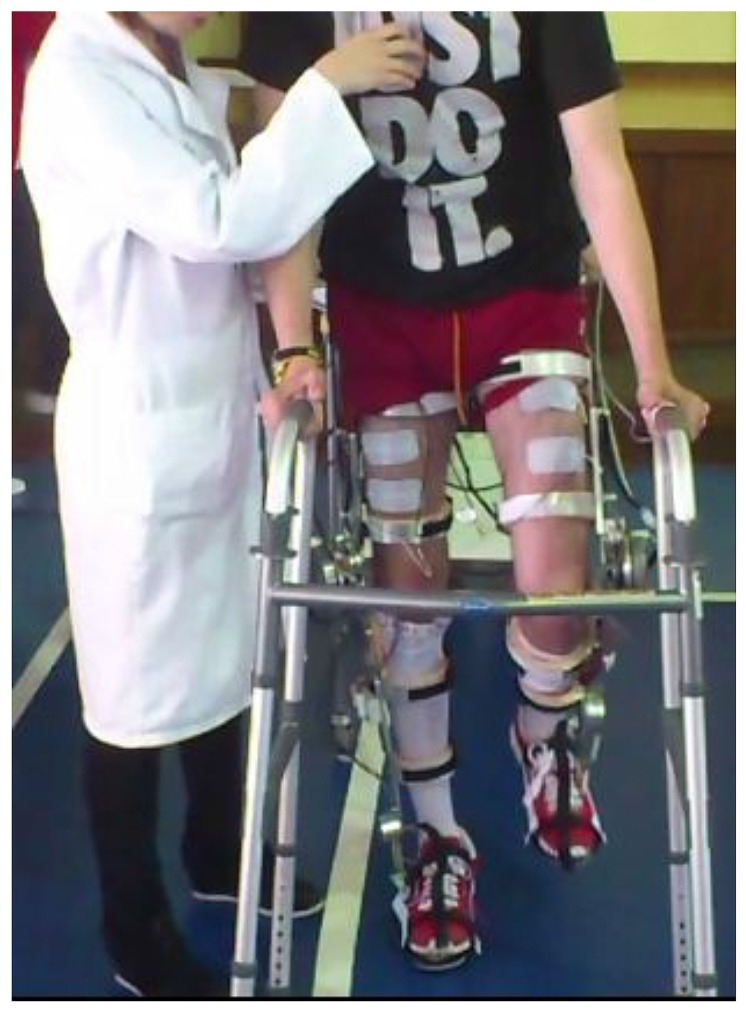
**Experimental setup for data collection and testing hybrid gait training with Kinesis overground robot**.

The first session consisted of a examination of response to EMS (S in **Figure 1**). The objectives for this session were to quantify the muscular response to the stimulation and also to get the patient used to the stimulation. Within this session, both flexor and extensor knee muscle groups of both legs were stimulated for 15 min. At the following session (T in **Figure 1**), the Kinesis device was introduced to the patients and learning exercises were carried out (**Figure 2**). The objective of this session was to train the patient to use the hybrid gait trainer and getting acquainted with the walking technique: bend to the side to lift the heel prior to initiate a step and then pressing the button. The Kinesis hybrid gait trainer was adjusted to the patient anthropometry within this session. The total walking time in this session did not exceed from 10 min. The remaining three sessions of the intervention were the actual hybrid walking experiments. As explained below, different configurations of the AAN controller were investigated in separate days (HC, CO, and HP in **Figure 1**, defined below).

During the second week (no intervention week) no intervention was administered. The objective of this design was to assess patient walking function before and after, and 1 week after the intervention with Kinesis (and associated experiments). For that purpose, assessment of gait function was performed prior-, post-, and 1 week after the hybrid walking intervention. The walking function assessment, denoted as EI, EII, and EIII in **Figure 1**, took place in separated days. The protocol was equal for all evaluation sessions, and was comprised by a MMT (Office, 1943) score of the sagittal plane, spasticity assessment by the Ashworth and PENN scales (Ashworth, 1964), and a 6mWT, where the time to cover the first 10 m was registered (10mWT). Patients used assistive devices for the walking conditions if regularly used to walk, and all conditions were assisted by a physiotherapist.

As introduced above, three configurations of the AAN controller have been included in the study (HC, CO, and HP in **Figure 1**). It is recalled here that Kinesis hybrid-cooperative controller is designed to modulate stimulation and robotic assistance during walking, which corresponds to the HC control configuration (hereinafter only configuration). In order to provide a better understanding of the performance of the hybrid system during overground gait training, two additional walking experimental conditions where designed (CO and HP in **Figure 1**), this is, to separately test the performance of the NP and the robotic components. The following codes are used to identify the walking experimental conditions:

– HC: hybrid-cooperative. During this condition EMS and robotic assistance are modulated during walking. The implementation of the controller used in this condition and its validation with healthy users presented elsewhere (del-Ama et al., 2013).

– CO: cooperative-only. During this condition the ILC was disabled, thus Kinesis can only bring adaptable robotic assistance to the patient during the swing phase stimulation of extensor muscles was not disabled to provide support during the stance phase.

– HP: hybrid-stiff. During this condition, the robotic assistance of Kinesis was held constant while the EMS controller operates similarly to HC configuration (learning and monitoring states enabled).

Each condition was tested in separated sessions to avoid fatigue-related effects. The conditions were tested under this sequence for each subject: HC-CO-HP. Measuring of BP and HR immediately prior and after the walking test were included for monitoring the physiological impact.

### DATA ANALYSIS

In order to evaluate the direct effects of the intervention we analyzed the gait function and the biomechanical performance during the HC conditions (results during the CO and HP conditions are not presented in the current study). Assessment of gait function was performed gathering data from the 6mWT and 10mWT, MMT for the lower limbs and Ashworth spasticity scale. Results were group averaged and compared across assessment points. Mean and standard deviation for, 10mWT and 6mWT were obtained. Kinesis performance was assessed in terms of actual knee angle, torque interaction, stimulator control output and torque field stiffness.

The normalized average stimulation output for knee extensor and flexor muscles (acronym NILC for flexor muscles) were calculated during the swing and stance phases respectively. This normalized average was calculated integrating the stimulator output during the walking phase (for swing and stance separately), and dividing the result by the maximum stimulation output theoretically achievable, which corresponds to a 450 μs saturated output for the entire walking phase. This normalized stimulation output gives a representative value є [0, 1] where 0 means no stimulation during the entire phase, and 1 means a constant, saturated stimulation output of 450 μs for the entire walking phase.

A VAS was used to assess user fatigue, pain, and comfort. The VAS consists of a 10 centimeters rectangle that is extensively used to assess perceived pain in clinical settings (DeVine et al., 2011). With this scale, the user rates the pain perception placing a mark inside the rectangle, rating from no pain at all at the left edge of the rectangle, to intolerable pain at the right edge of the rectangle. Measuring the distance from the left edge to the mark gives a value є [0, 10] that represents the user perceived pain, or fatigue and comfort in this protocol.

## RESULTS

### OVERGROUND ROBOTIC-GUIDED WALKING WITH NEURO-PROSTHETIC CONTROL (HC)

**FIGURE 3:**
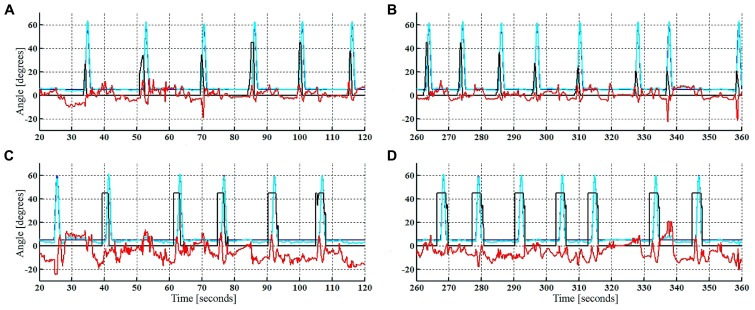
**Reference (blue) and actual (cyan) knee joint sagittal angle, interaction forces (red, [N.m/deg]), and ILC stimulation output (black, [μs]) during HC walking condition.** First steps of left leg **(A)**, last steps of left leg **(B)**, first steps of right leg **(C)**, last steps of right leg **(D)**.

**FIGURE 4:**
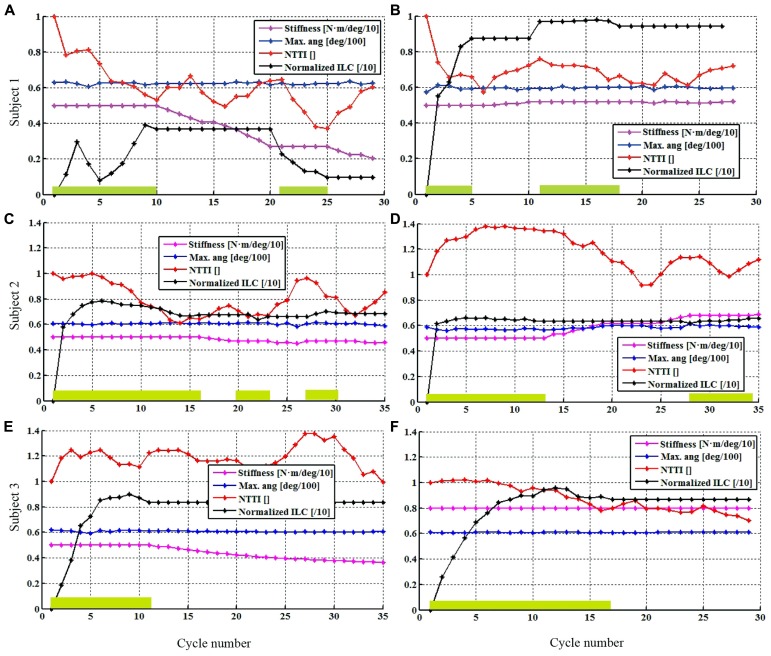
**Single-subject performance in the cycle domain during the HC condition.** Controller stiffness (magenta line), NTTI (red), maximum angle achieved during flexion (blue line), normalized torque-time integral (red line), and NILC (black line) of left **(A,C,E)** and right **(B,D,F)** legs. Green boxes indicate when EMS learning state was active within the cycle. Controller stiffness, maximum angle and normalized stimulation curves are scaled for visualization purposes.

**FIGURE 5:**
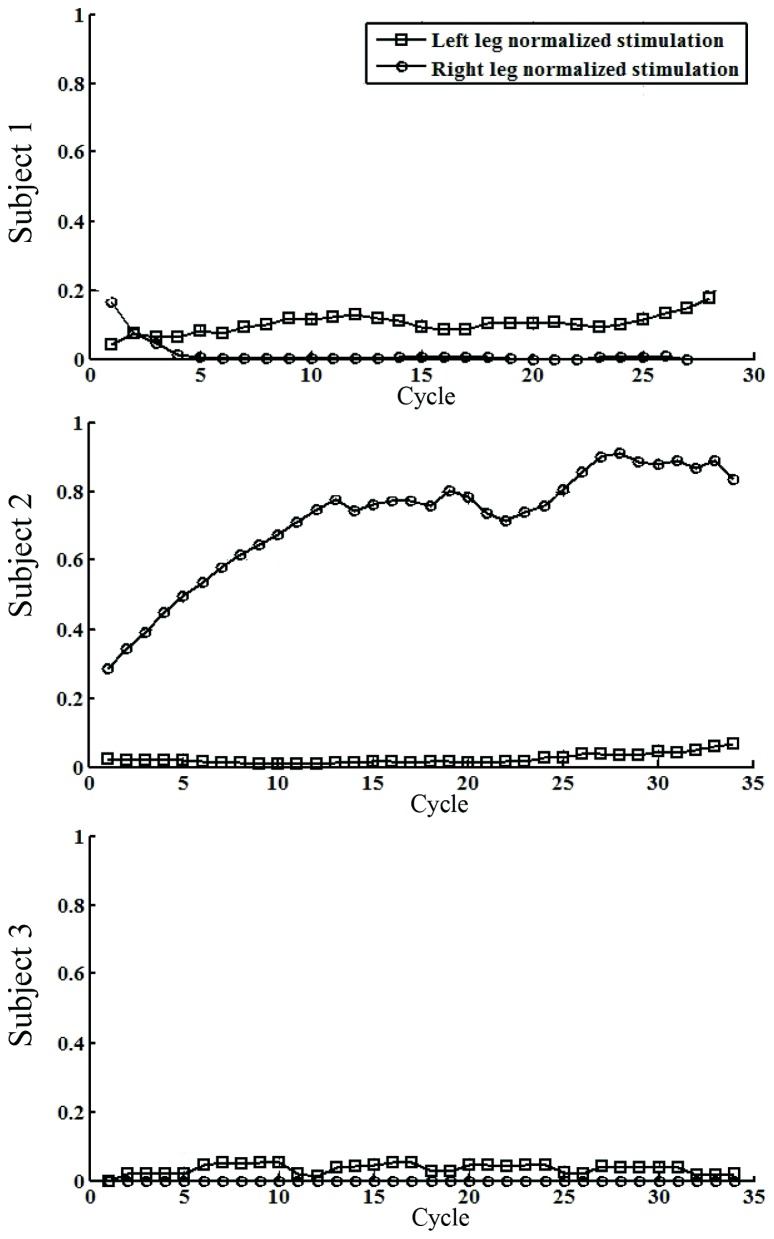
**Single-subject performance of quadriceps EMS during the walking experiment.** The data represents the averaged stimulation for the knee extensor muscles during the stance phases of walking. Cycle means number of step.

Overground walking performance was evaluated for the HC condition, with representative data from a single participant (Subject 1) with iSCI shown in **Figure 3**. Time-evolution of knee patterned and actual kinematics, interaction forces and the ILC stimulation output for the flexor muscles for both legs during walking with the HC, are represented. For this subject, knee kinematics of both legs were successfully controlled, and there were no dangerous situations reported during the experiment. Stimulation of left leg flexor muscles during swing (**Figures 3A,B**) was significantly lower than for the right leg (**Figures 3C,D**). A knee hyper-extension pattern at the right leg was obtained during the stance phase, maintained until prior swing phase (see actual knee joint angle, **Figures 3C,D**) and this hyper-extension was also evident in the measured interaction forces. On the contrary, extension (positive) interaction forces were observed during the swing phase of the right leg. Cycle-related performance during the HC experiment was analized for each subject, with data from participants with iSCI shown in **Figure 4**. The average normalized intensity of quadriceps EMS for the stance phases of the walking experiment were also analyzed for each subject, shown in **Figure 5**.

In Subject 1, differences in the functional status of both legs were observed (**Figures 4A,B**), coherent with the demand of EMS intensity and the stiffness of the exoskeleton. For the left leg, EMS intensity (represented in black) was lower when compared to the right leg muscles (maximum reached: 40% for the left, 97% for the right), and the first learning period required more steps. Interestingly, after the first muscle fatigue detection of the left leg (cycle 20), the second learning period led to a lower stimulation intensity, while the NTTI^1^ further decreased during the second monitoring period (cycles 25–29). This decrease could not be attributed to an effect of EMS, because the pulse duration was significantly low to produce muscle contraction (10% of NILC). NTTI was increased in the last five cycles of the walking trial, due to the augmentation of muscle fatigue. There was a marked reduction in the interaction force for both legs, up to a 40% of the initial TTI. The EMS was considerably high for the right leg, above the 80% of the maximum achievable stimulation, for most of the trial. Decay on muscle performance was detected at cycle 10, probably due to the fatigue produced by the high intensity of EMS that resulted in the first iteration period (**Figure 4B**, cycles 1–5). The second iteration required a longer period (cycles 11–18), achieving a similar reduction in TTI. An increase on TTI was observed for the left leg for the last five cycles of the trial. The stiffness of the left knee joint was progressively reduced during the monitoring periods, while the stiffness of the right knee joint could not be reduced, although the stimulation was high and a reduction in NTTI was achieved.

In the case of Subject 2, the performance in the cycle domain during the HC condition is presented in **Figures 4C,D**. In particular, the obtained EMS patterns are summarized. NILC for both legs achieved the 70% of the maximum achievable stimulation intensity during the swing phase. After the first learning period of the EMS controlling the right leg, a decrease on NTTI is observed along with an increase on joint stiffness. Muscle fatigue was detected in cycle 27 which activated a second monitoring state. A more moderate increase was required EMS intensity for the left leg during the first learning period, reaching similar equivalent right leg EMS intensity, and related to the observed decrease on NTTI. After the learning period, the stiffness was slightly reduced.

In the case of Subject 3, the performance in the cycle domain during HC walking condition is presented in **Figures 4E,F**. Left knee joint interaction forces were of low magnitude during the stance phase. The intensity of EMS was lower if compared to the right leg (85% of the maximum achievable intensity, black line) after the learning prior. The stiffness decreased with a minor decay during the monitoring period. Stimulation of right knee flexor muscles exhibited a saturated pattern from the first cycles the swing phase, as was observed in the cycle domain, where the NILC for the right leg (after the first learning period) reached approximately 100% of the maximum achievable EMS (**Figure 4F**). The NTTI shown a decrease (red line) after cycle 10. Stiffness increased during the monitoring period (magenta line), as expected.

**FIGURE 6:**
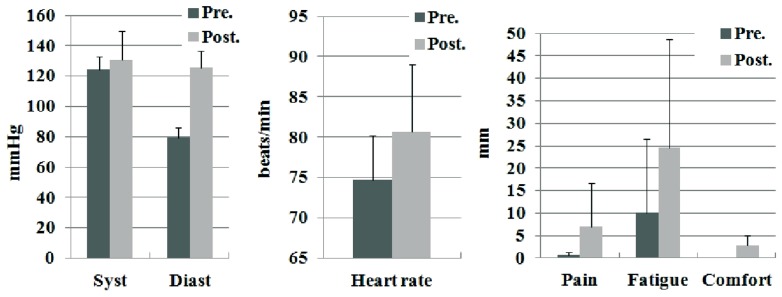
**Pre and post condition measures of physiological effort and subjective perceptions.** The reported data are average and standard deviation for systolic BP, diastolic BP, heart rate, pain, fatigue, and comfort.

Physiological effort data (**Figure 6** and **Table 2**) shows that the HC condition resulted in an increase in the systolic and diastolic BP after the experiment. Subjective perception of fatigue, pain and comfort were rated under the 50% of the scale for across all conditions (**Figure 6** and **Table 2**).

### IMPACT ON WALKING FUNCTION

Evaluations of gait functionality were conducted prior, after, and 1 week after the intervention week (**Figure 1**). The results are presented in terms of relative changes on the outcome measures. These walking tests were conducted with diverse types of external support (patients 1 and 2 without external aids, patient 3 used a walker). **Table 1** shows the relative changes on the time needed to reach 10 m, and the distance covered in 6 min. The data revealed significant improvements after week I–II with less time required to complete 10 m and covered more distance in 6 min. One week after intervention, the patients further improved on gait function (II–III), but overall this differential improvement was smaller than post intervention I–II. The improvement of the intervention I–II was sustained and augmented at the final evaluation (I–III).

**Table 1:** Pre–post condition results of 10mWT an 6mWT tests during the experimental conditions.

	I–II	II–III	I–III
10mWT [sec]	-13.6 ± 28.2	-16.2 ± 33.5	-29.8 ± 61.7
6mWT [m]	44.2 ± 59.3	17.8 ± 21.4	62.0 ± 79.6

*The reported data are average and standard deviation.*

**FIGURE 7:**
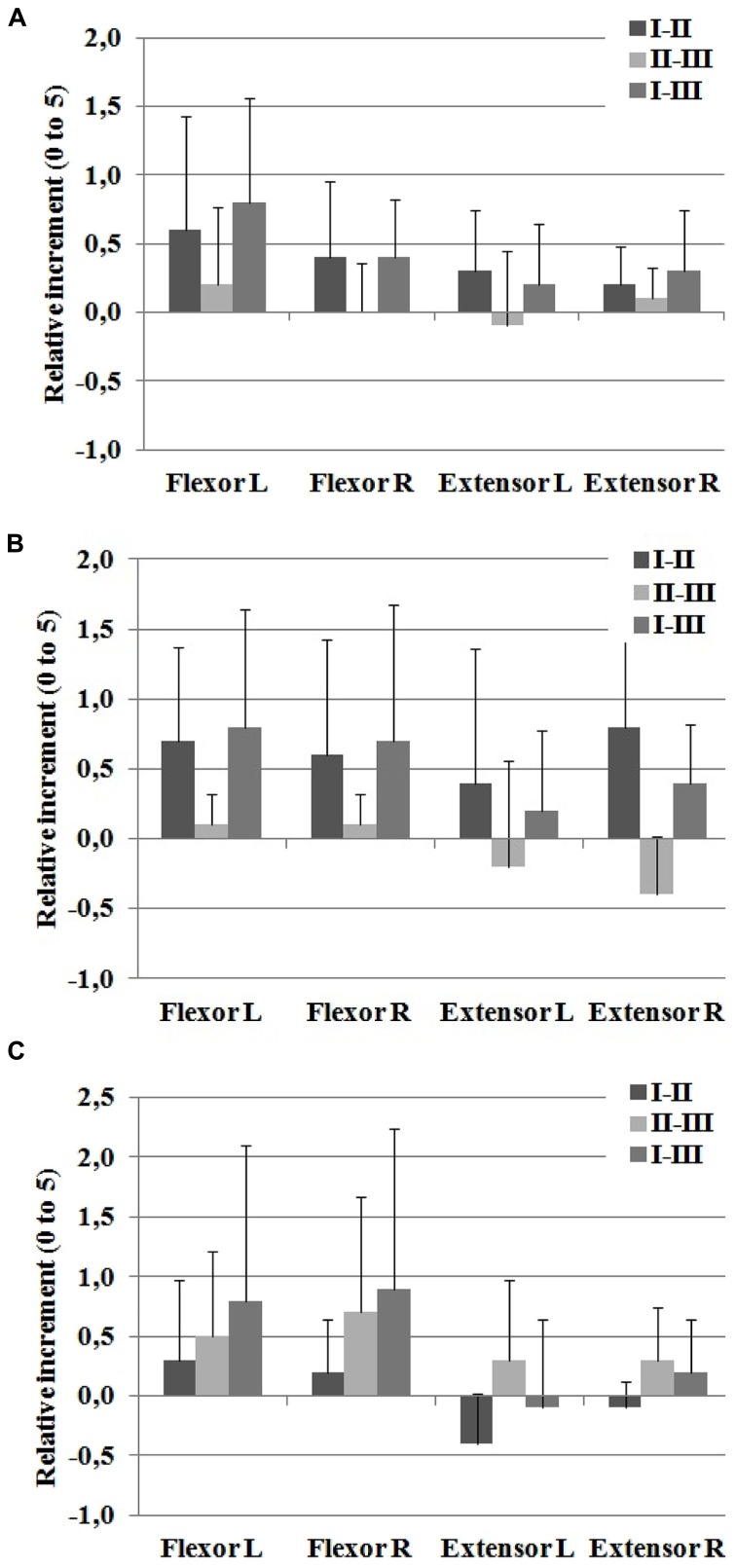
**Sagittal manual muscle testing (MMT) score for (A) hip, (B) knee, and (C) ankle joints.** The reported data are average and standard deviation of the relative changes between the examination sessions I–II, II–III, and I–III.

Muscle examination and grading strength (MMT) was performed after the interventions and revealed significant improvements on muscles controlling hip and knee joints. Data from the group of patients are provided in **Figure 7** and **Table 2** with MMT scores for hip, knee and ankle joints in the sagittal plane. In particular, results show that highest increments on hip MMT score were found for the I–II intervention week, sustained until the I–III intervention week (**Figure 7A** and **Table 2**). The increase on hip MMT can be explained to the effort required to move the extra weight of the exoskeleton, whilst no assistance was provided at hip joint level. A greater impact was observed on the muscle strength of flexor muscles than on extensor muscles. It can hypothesized that the effort to extend the hip be lower as result of the passive hip extension produced as consequence of contralateral leg flexion combined with trunk forward lean. Data also revealed increments in knee flexor and extensor muscle groups (intervention week I–II). In particular, higher improvement in knee extensor muscles was observed in session week II–III, and on the contrary, the flexor muscles shown a relative decrement on MMT for this intervention. When the knee was passively driven by the exoskeleton, no increments on knee muscular MMT would be obtained. For the knee flexor muscles, decreased MMT score was observed during session week II–III, which may be explained by a significantly lower demand during standard rehabilitation exercises in comparison with the hybrid walking condition. As a result, the increase on the flexor MMT score was not sustained in week II–III.

**Table 2:** Summary of evaluation results: 10mWT and 6mWT tests and subjective scales.

	HC
10mWT [sec]	3.3 ± 1.6
6mWT [m]	17.0 ± 20.2
Systolic BP [mmHg]	6.7 ± 20.8
Diastolic BP [mmHg]	7.3 ± 15.0
Hear rate [beat/min]	6.0 ± 14.6
Pain [cm]	6.3 ± 17.3
Comfort [cm]	2.9 ± 2.1
Fatigue [cm]	14.7 ± 50.5

*Data provided for fatigue, pain, BP and HR are pre–post relative increment. The reported data are average and standard deviation.*

For the ankle flexor muscles (**Figure 7C**), increased MMT score was observed after I–II and II–III weeks, which was sustained after 1 week (week I–III). The increased scores were higher for the no-intervention week (II–III vs. I–II). Ankle extensor muscles revealed a decrease after the I–II intervention week, but were increased after the II–III non-intervention week (**Table 2**). A hypothesis for this finding is that the spring-driven ankle actuator of the exoskeleton, which supported the foot during the swing phase, could have reduced the ankle extensor muscle ability. This phenomenon has been described previously in experiments with healthy subjects walking with a pneumatic KAFO-type exoskeleton (Kao et al., 2010). Nevertheless, other variables not related to the study may have influence on these results. Finally, a single-subject difference in articular knee joint range after the intervention session I–II with an increase on the knee joint range of movement in patient 2 (left knee ROM: 10° extension, 110° flexion; right knee: 5° extension, 120° flexion).

**FIGURE 8:**
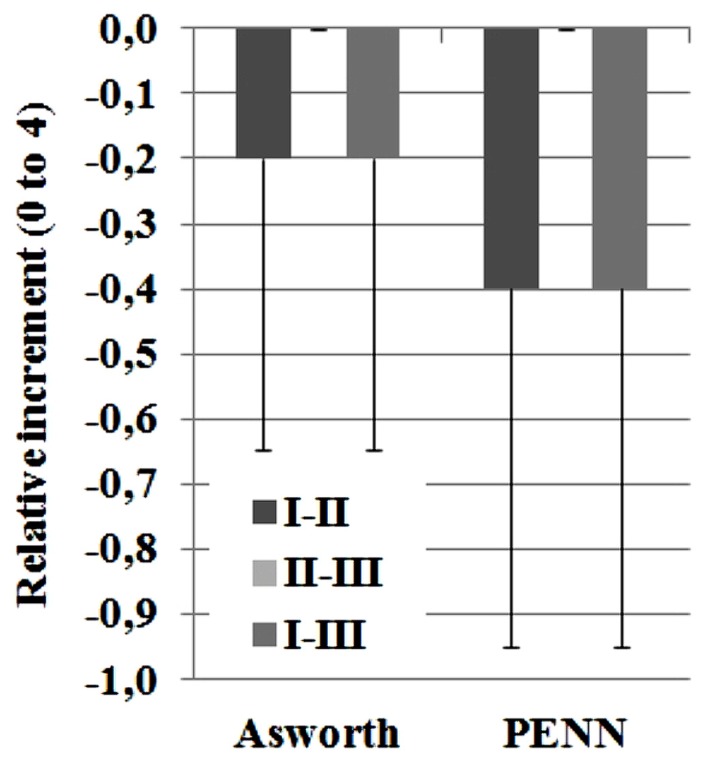
**Results for spasticity ASHWORTH and PENN scales.** Score can range from 0 to 4. The reported data are median and range.

Analysis of spasticity measures after hybrid gait training revealed marked differences in the ASHWORTH and PENN scales for measuring spasticity (**Figure 8**) for all subjects. Average relative increments in Ashworth scale was -0.2 ± 0.4 and Penn spasm frequency scale was -0.4 ± 0.5 after the intervention week.

## DISCUSSION

The purpose of this study was the assessment of the effects of the overground gait therapy by means of overground exoskeletons with neuro-prosthetic control. This analysis could be useful to provide the clinicians with objective data for interpretation of the appropriateness of this methodology as part of the treatment for the patient population. As such, evaluation of biomechanical and functional variables during and after hybrid gait training in these conditions provides insight into the response of iSCI patients to such treatment. Only few previous studies including NP technology and wearable exoskeletons provided preliminary insight into the potential outcome of such overground training paradigm in the promotion of a locomotor pattern (Esquenazi et al., 2012; Zeilig et al., 2012).

### EFFECTS IN GAIT PERFORMANCE

Evaluation of the participants’ response revealed that tested hybrid walking intervention as a therapy was tolerated by the patients; no adverse effects were produced and the physical demand was tolerable. The participants were able to complete 6 min of walking with the system after 1 day of practice. The improvement in the 6mWT, the 10mWT, lower limb MMT and spasticity indexes demonstrate that walking function of the patients was improved after the intervention with the hybrid trainer. The hybrid control system adapted to the residual function of the participants during the task, modulating EMS and robotic assistance as needed. The observation of perceived pain, fatigue and comfort scores for the HC configuration revealed that training with Kinesis led to values on average were below 0.5. This provides an innovative indication of the positive tolerance of the participants to the proposed hybrid walking therapy. Our previous review of the literature did not trow explicit results of this evaluation but some specific claims on the lack of pain perception (Goldfarb et al., 2003), and improved feeling of safety have been reported (Popovic et al., 1989). In comparison with the reported results on perception of robotic-assisted walking (Esquenazi et al., 2012; Zeilig et al., 2012), our results are equivalent in terms of pain but with lower scores in relation to fatigue.

### HUMAN–ROBOT INTERACTION DEMANDS FOR HYBRID GAIT TRAINING

From the perspective of assisted gait training, shortening the preliminary training period that is required to start a robot-aided treatment is important for the key components of the recovery after neurological damage, such as active engagement, motivation and patient-cooperative control. It is to be noted that the participants in this study were able to use the system as an overground robot-aided treatment after 1 day of practice (session T in **Figure 1**). This is significantly lower to requirements reported with the Rewalk, 25 days (average) needed by the patients to achieve autonomous use of Rewalk for an equivalent period of time (5–10 min; Esquenazi et al., 2012; Zeilig et al., 2012). This is important from a clinical perspective, although clear differences between Kinesis and Rewalk systems, the evaluation protocols and patient conditions should be considered when comparing requirements across devices. Rewalk controller demands patients to learn a strategy to trigger gait initiation and stopping by tilting the trunk. With this function, patients are required to learn a strategy to drive the Rewalk, maintaining balance with the help of walking crutches. With the Kinesis system used in the present study, the patient triggers each step when feeling table and ready to keep stepping, and also, the walker aid during the training condition provides a more stable support than crutches. These key differences can be reflected when comparing the results for 10mWT and 6mWT. On the one hand, Kinesis hybrid gait training led in average to double time to walk 10 m, and half distance covered in 6 min than Rewalk. On the other hand, Kinesis sequential manual trigger of stepping allowed the patients to efficiently drive the after a single day training session. This is important to bring the hybrid therapy early to the patient, avoiding long training periods as reported for gait neuroprosthetics (Thrasher et al., 2006). Human–robot interfaces to drive walking control with robotic exoskeletons for training require more research and development to more naturally operate the device without increasing the required training time an maintaining safety (Quintero et al., 2012).

### ASSIST-AS-NEEDED HYBRID GAIT TRAINING

The extent of cooperative assistance provided by the hybrid robot is a key factor that may contribute to observed differences between treatment groups. Evaluations of the control-cooperative and AAN features of the hybrid gait trainer reveal, in our opinion, significant advances in the state-of-the art of control approaches for hybrid exoskeletons. First, the proposed hybrid controller allowed adapting the artificial muscular stimulation patterns to the neuromuscular status and performance of the patient. The hybrid controller showed to efficiently deliver stimulation as-needed to knee flexor and extensor muscles as a function of gait phase and voluntary contribution. Accordingly, customized stimulation patterns are delivered for each muscle of each leg to stably drive the knee joints. Across individuals, the dynamically obtained EMS patterns varied muscle activation from no stimulation (e.g., Subject 1) to maximum stimulation (e.g., Subject 3) to specific functional requirements during stance or swing phases. Finally, these results supports close-loop muscle stimulation from a functional standpoint. However, the closed-loop control of muscle stimulation tested in the current study does not optimally manage the relationship between intensity of the EMS and force production. The results show that for a number of gait cycles EMS within the hybrid controller was not able to produce a significant muscular contraction for any normalized stimulation intensity below the 20%. Moreover, the opposite situation was also noticed in a number of trials, characterized by a saturated output of the EMS controller that could not produced a muscle contraction (e.g., Subject 3, leg knee flexor muscles). These effects constitute inherent limitations of the hybrid control system, which is not able to map the non-linear relationship between muscle stimulation and force production. Such feature in future controllers of hybrid gait exoskeletons would be important to provide a criteria for more efficient modulation of EMS regarding the range of muscle activation of targeted muscles.

Muscle fatigue estimation in hybrid exoskeletons has been addressed by off-line methods, such as the isometric recruitment curve (Goldfarb et al., 2003). The muscle performance monitor within the hybrid gait controller relies on leg-exoskeleton physical interaction as feedback for modulation control output. This scheme is expected to contribute to delays the appearance of muscle fatigue. However, the current results indicate that voluntary leg movements influence the physical interaction, with an effect on the accuracy of muscle fatigue estimation. Two mechanisms can explain the effects on the physical interaction. First, the varying volitional contribution to joint force provided by the subject. In this case, patient slacking would lead to false detection of muscle fatigue. Second, the method for online normalization of the FTI values that could lead to low accuracy in FTI estimation. This suggests that more robust methods to uncouple patient contribution to the movement from EMS performance and robot assistance are needed to improve muscle fatigue management and therefore optimize patient volitional contribution.

The impact of the hybrid walking therapy on patient gait abilities was evaluated. Greater improvements in gait function, sagittal MMT score, and spasticity, for the intervention week I–II was concluded with the observation week II–III. There are some limitations to this study. First, the sample size was low, and with heterogeneous lesions, therefore we recommend caution when generalizing the study results. Second, we tested walking conditions to reveal effects of the hybrid approach in a non-randomized testing order of configurations due to practical considerations. As a result, this may led to uncontrolled learning effects. A third limitation is that we did not test the effect of intensity and long-term retention of hybrid gait training. However, these results encourage the design of further studies that would allow knowing whether iSCI can retain the functional gains over longer time periods as a function of treatment dose.

In summary, participants tolerated the hybrid gait intervention delivered by Kinesis. The patients were able to complete 6 min of walking with the system after 1 day of practice. Mutual adaptations were observed between the patient and the system that were assessed through the analysis of the physical interaction. The hybrid-cooperative control was able to compensate bilateral pathologic walking patterns by autonomously increasing the stimulation of the knee joint muscles and increasing the displayed stiffness of the robotic actuators. The improvement in the 6mWT, the 10mWT, lower limb MMT, and spasticity indexes demonstrate this training paradigm, termed hybrid gait training, to improve short-term locomotor performance that is consistent with promoted recovery of neuromuscular control for gait. The results of the current pilot study provide proof-of-concept for the feasibility of combining neuroprosthesis and actuated exoskeletons as tools for robotic training of gait function in motor incomplete SCI patients, guaranteeing and motivating further research in the field. Further work is required to optimize several open questions of hybrid gait control, such as assessment of volitional muscle contribution during EMS and optimization of muscle fatigue detection and quantification. Furthermore, testing the hybrid strategies on multiple joints will be interesting to cope with requirements of wider patient populations. Also, research is required to elucidate the changes in neuromuscular activity of targeted lower limb muscles during and after the intervention and to elucidate the mechanisms that may explain a recovery of neural control. The reduced number of subjects studied and the small amount of training time (6 min for the walking experiments T, HC, CO, and HP) limits extrapolating the results of the hybrid walking therapy to the incomplete SCI population. Besides, the heterogeneity of the functional status of the patients can be also regarded as a limitation in the study design. While this is true for elucidating the effects of the hybrid training, the results showed the ability of Kinesis system and AAN controller to adapt to such different functional conditions. In conclusion, future clinical trials are required to establish the long-term therapeutic benefits of overground hybrid training in restoring gait function in a wide and more representative population of incomplete SCI subjects.

## Conflict of Interest Statement

The authors declare that the research was conducted in the absence of any commercial or financial relationships that could be construed as a potential conflict of interest.

## Footnotes

1 Normalized time integral of the interaction torque during the swing phase.

## ABBREVIATIONS

6mWT: 6 minutes walking test
10mWT: 10 meters walking test
AAN: assist-as-needed
BP: blood pressure
CO: cooperative-only condition
EMS: electrical muscle stimulation
FSR: force sensing resistor
FTI: force time integral
HC: hybrid-stiff condition
HGO: hip guidance orthosis
HKAFO: hip knee ankle foot orthosis
HP: hybrid-cooperative condition
HR: heart rate
ILC: iterative learning controller
iSCI: incomplete spinal cord injury
KAFO: knee ankle foot orthosis
MFE: muscle fatigue estimator
MMT: manual muscle test
NP: neuroprosthetic
NTTI: normalized torque-time integral
PWM: pulse width modulation
QUEST: Quebec user evaluation of satisfaction with assistive technology
RMS: root mean square
VAS: visual analog scale
WISCI II: walking index for spinal cord injury version 2

## References

[B1] Ashworth B. (1964). Preliminary trial of carisoprodol in multiple sclerosis. *Practitioner* 192 540–542 14143329

[B2] Creasey G. H. Ho C. H. Triolo R. J. Gater D. R. DiMarco A. F. Bogie K. M. (2004). Clinical applications of electrical stimulation after spinal cord injury. *J. Spinal Cord Med.* 27 365–375 15484667 10.1080/10790268.2004.11753774

[B3] del-Ama A. J. Bravo-esteban E. Moreno J. C. Gómez-soriano J. Koutsou A. D. Gil-agudo Á. (2012a). “Knee muscle fatigue estimation during isometric artificially elicited contractions in incomplete spinal cord injured subjects,” in *Proceedings of the 2012 International Conference on Neurorehabilitation (ICNR2012): Converging Clinical and Engineering Research on Neurorehabilitation* (Berlin: Springer) 329–333

[B4] del-Ama A. J. Koutsou A. D. Moreno J. C. (2012b). Review of hybrid exoskeletons to restore gait following spinal cord injury. *J. Rehabil. Res. Dev.* 49 497–514 10.1682/JRRD.2011.03.0043 22773254

[B5] del-Ama A. J. Koutsou A. D. Moreno J. C. De-los-Reyes A. Gil-Agudo A. Pons J. L. (2012c). Review of hybrid exoskeletons to restore gait following spinal cord injury. *J. Rehabil. Res. Dev.* 49 497–514 10.1682/JRRD.2011.03.0043 22773254

[B6] del-Ama A. J. Moreno J. C. Gil-Agudo A. De-los-Reyes A. Pons J. L. (2012d). Online assessment of human-robot interaction for hybrid control of walking. *Sensors* 12 215–225 10.3390/s120100215 22368465 PMC3279209

[B7] del-Ama A. J. Gil-agudo Á. Pons J. L. Moreno J. C. (2013). Hybrid FES-robot cooperative control of ambulatory gait rehabilitation exoskeleton. *J. Neuroeng. Rehabil.* 11 27 10.1186/1743-0003-11-27 PMC3995973 24594302

[B8] DeVine J. Norvell D. C. Ecker E. Fourney D. R. (2011). Evaluating the correlation and responsiveness of patient-reported pain with function and quality-of-life outcomes after spine surgery. *Spine (Phila Pa 1976).* 36 S69–S74 10.1097/BRS.0b013e31822ef6de 21897347

[B9] Dollar A. M. Herr H. (2007). Active orthoses for the lower-limbs: challenges and state of the art. *IEEE Int. Conf. Rehabil. Robot.* 1 968–977

[B10] Dollar A. M. Herr H. (2008). Lower extremity exoskeletons and active orthoses: challenges and state-of-the-art. *IEEE Trans. Robot.* 24 144–158 10.1109/TRO.2008.915453

[B11] Esquenazi A. Talaty M. Packel A. Saulino M. (2012). The ReWalk powered exoskeleton to restore ambulatory function to individuals with thoracic-level motor-complete spinal cord injury. *Am. J. Phys. Med. Rehabil.* 91 911–921 10.1097/PHM.0b013e318269d9a3 23085703

[B12] Goldfarb M. Durfee W. K. (1996). Design of a controlled-brake orthosis for FES-aided gait. *IEEE Trans. Rehabil. Eng.* 4 13–24 10.1109/86.486053 8798068

[B13] Goldfarb M. Korkowski K. Harrold B. Durfee W. (2003). Preliminary evaluation of a controlled-brake orthosis for FES-aided gait. *IEEE Trans. Neural Syst. Rehabil. Eng.* 11 241–248 10.1109/TNSRE.2003.816873 14518787

[B14] Graupe D. Cerrel-Bazo H. Kern H. Carraro U. (2008). Walking performance, medical outcomes and patient training in FES of innervated muscles for ambulation by thoracic-level complete paraplegics. *Neurol. Res.* 30 123–130 10.1179/174313208X281136 18397602

[B15] Hesse S. Waldner A. Tomelleri C. (2010). Innovative gait robot for the repetitive practice of floor walking and stair climbing up and down in stroke patients. *J. Neuroeng. Rehabil.* 7:30 10.1186/1743-0003-7-30 PMC2914004 20584307

[B16] Kao P.-C. Lewis C. L. Ferris D. P. (2010). Short-term locomotor adaptation to a robotic ankle exoskeleton does not alter soleus Hoffmann reflex amplitude. *J. Neuroeng. Rehabil.* 7:33 10.1186/1743-0003-7-33 PMC2917445 20659331

[B17] Lam T. Wolfe D. Eng J. J. Domingo A. (2010). *Lower Limb Rehabilitation Following Spinal Cord Injury*, Version 4, Vol. 3. Vancouver, BC: Spinal Cord Injury Rehabilitation Evidence

[B18] Nightingale E. J. Raymond J. Middleton J. W. Crosbie J. Davis G. M. (2007). Benefits of FES gait in a spinal cord injured population. *Spinal Cord* 45 646–657 10.1038/sj.sc.3102101 17646840

[B19] Office H. M. S. (ed.) (1943). *Aids to the Investigation of Peripheral Nerve Injuries*. London: Medical Research Council (Great Britain), Nerve injuries Committee

[B20] Popovic D. Tomovic R. Schwirtlich L. (1989). Hybrid assistive system-the motor neuroprosthesis. *IEEE Trans. Biomed. Eng.* 36 729–737 10.1109/10.32105 2787281

[B21] Quintero H. A. Farris R. J. Ha K. Goldfarb M. (2012). Preliminary assessment of the efficacy of supplementing knee extension capability in a lower limb exoskeleton with FES. *Conf. IEEE Eng. Med. Biol. Soc.* 2012 3360–3363 10.1109/EMBC.2012.6346685 PMC3688042 23366646

[B22] Stauffer Y. Allemand Y. Bouri M. Fournier J. Clavel R. Metrailler P. (2009). The WalkTrainer – a new generation of walking reeducation device combining orthoses and muscle stimulation. *IEEE Trans. Neural Syst. Rehabil. Eng.* 17 38–45 10.1109/TNSRE.2008.2008288 19211322

[B23] Thrasher T. A. Flett H. M. Popovic M. R. (2006). Gait training regimen for incomplete spinal cord injury using functional electrical stimulation. *Spinal Cord* 44 357–361 10.1038/sj.sc.3101864 16249784

[B24] Zeilig G. Weingarden H. Zwecker M. Dudkiewicz I. Bloch A. Esquenazi A. (2012). Safety and tolerance of the ReWalk^TM^ exoskeleton suit for ambulation by people with complete spinal cord injury: a pilot study. *J. Spinal Cord Med.* 35 96–101 10.1179/2045772312Y.0000000003 22333043 PMC3304563

